# A Mysterious Rash Following Cardiothoracic Surgery: A Not So Sweet Ending

**DOI:** 10.7759/cureus.69048

**Published:** 2024-09-10

**Authors:** Ahmad Daoud, Ronak Rajani, Vitaliy Androshchuk, Natalie Montarello

**Affiliations:** 1 Cardiology, Guy's and St Thomas' NHS Foundation Trust, London, GBR

**Keywords:** atypical rash, surgical replacement of valve, myelodysplastic syndromes, sweet's sydrome, infective endocarditis

## Abstract

A male in his 70s, with a recent history of aortic valve replacement, mitral valve repair, and permanent pacemaker implantation (PPM), developed a fever, raised inflammatory markers, and a disseminated rash. Despite being attributed a diagnosis of an unspecified connective tissue disorder and erythema nodosum at his local hospital, his symptoms continued to deteriorate. A subsequent urgent admission was arranged to his original cardiothoracic centre for the exclusion of infective endocarditis (IE). Although this was subsequently ruled out by echocardiography and microbiological evaluation, a diagnosis of Sweet syndrome (SS) was made following a punch biopsy of a skin lesion. This was later attributed to myelodysplastic syndrome following a bone marrow biopsy. In this report, we firstly describe our diagnostic algorithm for reaching this diagnosis and the characteristic skin lesions associated with this condition. We furthermore review the history of SS, its known associations, and treatment options.

## Introduction

The association of Sweet syndrome (SS) with infection and inflammation is well described and constitutes one of the minor criteria for the diagnosis of the condition [1]. We report a case wherein infective endocarditis (IE) in a high-risk individual was considered the likely associated disease entity at the onset of SS. While IE should be excluded in these patients, an association of SS with underlying occult malignancy, both haematologic and solid organ, is more likely. An awareness of this may enable clinicians to reach an earlier diagnosis and implement appropriate treatment.

## Case presentation

A male in his 70s was referred to our tertiary care centre with a fever and a generalized rash. His background history included a permanent pacemaker implantation (PPM) and a recent bioprosthetic aortic valve replacement (AVR) with mitral valve repair. Two months following cardiac surgery, a C-reactive protein (CRP) level was found to be over 100 mg/L, with no obvious infectious foci identified. One month later, he was also found to have an abnormal skin rash with tender papules, which was attributed to a diagnosis of erythema nodosum. He was started on low-dose systemic prednisolone, which resulted in a transient improvement in his symptoms before a further deterioration. This prompted a referral to a rheumatologist. Here he was found to have an isolated elevation in his anti-nuclear antibody (ANA) levels and a diagnosis of an undifferentiated connective tissue disorder was also issued. Despite being commenced on a short course of hydroxychloroquine, his systemic illness persisted.

Following two months of intermittent skin lesions, widespread rash, low-grade temperature spikes, and generalized fatigue, a decision was made to admit the patient back to his host cardiac centre to rule out IE and investigate his symptoms further.

**Figure 1:**
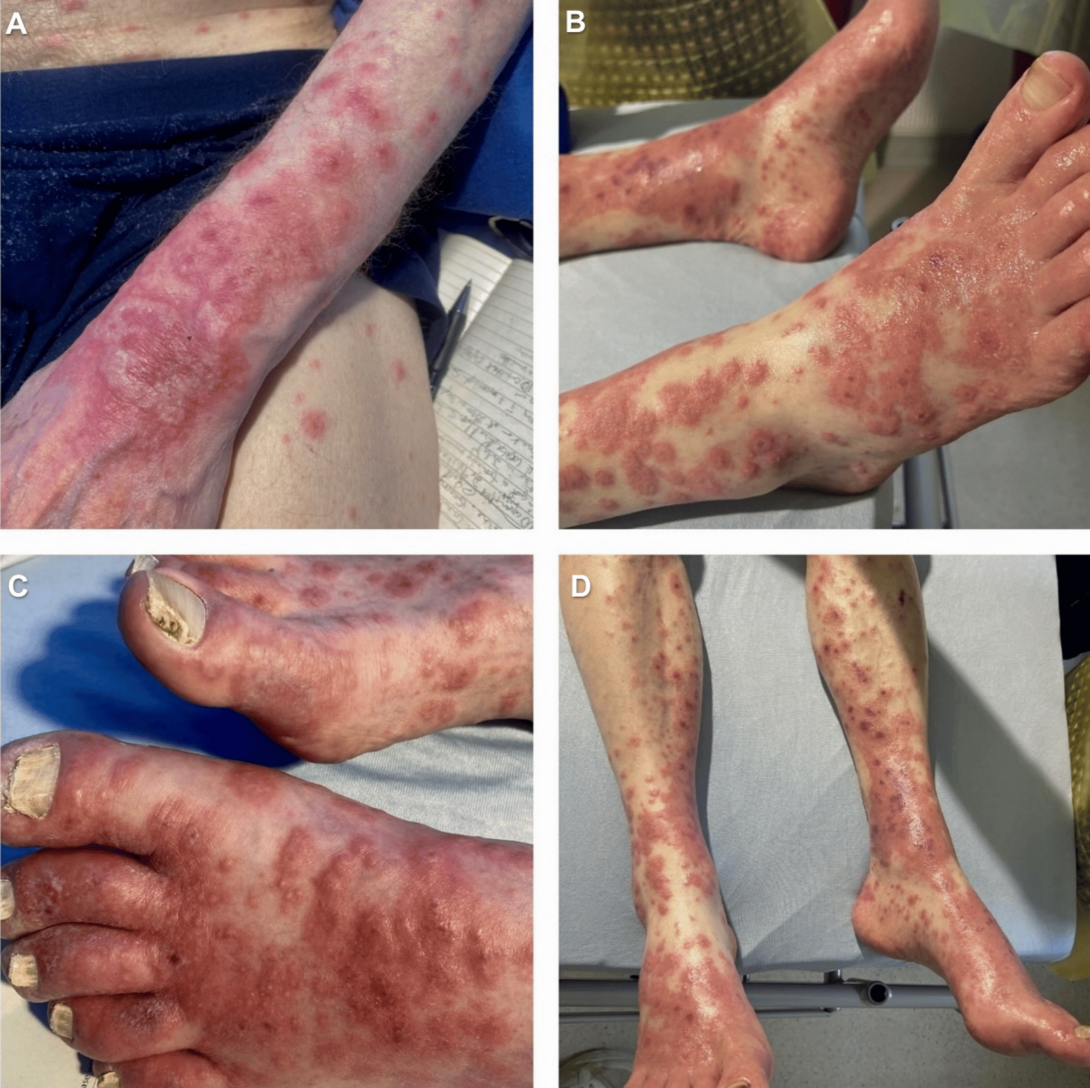
Widespread erythematous rash on initial presentation Macules and papules with coalescence into plaques and bullous formation centrally. (A) dorsum of the left hand and forearm and (B-D) lower limbs.

Upon admission, the patient reported symptoms of a low-grade fever accompanied by fatigue, night sweats, chills, rigours, and arthralgia. A clinical examination revealed no peripheral stigmata of IE and normal heart sounds. A note was made, however, of a striking generalised skin rash (Figure 1). This was painful, non-pruritic, and erythematous, and involved the trunk, extremities, and face. In the peripheries, there were coalescing targetoid lesions that were dusky in appearance but with no active blistering. Centrally, the lesions were more papulonodular. While the patient’s systemic steroids were continued, serial blood cultures were taken to exclude concomitant infection, and further referrals to dermatology and rheumatology were initiated.

Investigations

Laboratory results demonstrated a markedly raised CRP level of 350 mg/L. The white blood cell count was elevated to 21 x 10^9^/L with neutrophilic predominance (18.30 x 10^9^/L) and normal range eosinophil count. Multiple blood cultures were negative. Viral serology was normal.

Transthoracic echocardiography (TTE) revealed a bright, mobile, linear structure attached to chordae adjacent to the posterior mitral valve leaflet measuring 1.3 cm x 0.6 cm. Both the bioprosthetic aortic valve and the repaired mitral valve functioned normally. The findings were consistent with the post-operative echocardiogram and were not felt to be significant.

The rheumatology team arranged for an urgent full autoimmune panel which revealed solely positive ANAs with 1/80 antibodies titre. This slightly elevated ANA with negative double-stranded DNA (dsDNA) and extractable nuclear antigen (ENA ) was felt to be of doubtful clinical significance in the absence of symptoms of autoimmune connective tissue disease.

Due to a persistent rash, ongoing fever, and new-onset intermittent mild confusion, computed tomography (CT) of the chest, abdomen, and pelvis and magnetic resonance imaging (MRI) of the head were performed, which were unremarkable. 

Following four days of empirical treatment with low-dose prednisolone, acyclovir, and amoxicillin the patient experienced no significant improvement in his inflammatory markers or his systemic well-being. This led to a punch biopsy of a skin lesion being performed on day five of admission. This revealed dense dermal infiltrates composed predominantly of neutrophils with no evidence of leukocytoclastic vasculitis. This was consistent with a diagnosis of neutrophilic dermatosis or SS. Consequently, acyclovir and amoxicillin were stopped, and systemic high-dose prednisolone was commenced.

Differential diagnosis 

In the setting of a recent surgical aortic valve replacement and with a history of prior mitral valve repair and PPM implantation, IE was initially considered the most likely presenting diagnosis. This was ultimately discounted as the patient did not meet the Duke’s criteria [2]. Particularly, he had negative blood cultures, normal valve function on TTE, and no evidence of abnormal valve morphology or evidence of perivalvular complications of IE seen on CT. The abnormal linear mitral valve echogenicity noted on TTE was attributed to redundant chordae rather than vegetation.

Vacuoles, E1 enzyme, X-linked, autoinflammatory, somatic (VEXAS) syndrome, caused by a somatic mutation in haemopoietic progenitor cells, was also considered. This condition results in fever, inflammation, and haematologic symptoms predominantly in men in later life [3]. Genetic testing looking for a UBA1 gene mutation, however, was negative and excluded the diagnosis. 

Behçet’s disease, a chronic inflammatory condition that develops secondary to abnormal immune response and causes a plethora of symptoms including urogenital sores, generalized skin lesions, arthralgia, eye inflammation, as well as numerous systemic symptoms, may also manifest with cutaneous skin lesions and constitutional symptoms resembling those in SS [4]. This differential was also excluded owing to the clinical presentation.

Once a diagnosis of SS had been established, consideration was given as to whether this was medication or malignancy-related (Table 1). Although amiodarone was considered a potential trigger, this has not previously been convincingly found to be causal, and there was a poor temporal relationship between its commencement and clinical presentation. Therefore, it was not felt to be causal. Haematologic rather than solid organ malignancies are predominantly linked to SS. Therefore, owing to the whole-body CT being normal, a bone marrow aspirate and trephine biopsy were performed.

**Table 1:** Causes and associations of Sweet syndrome

Category	Examples					
Malignancies	Haematological: Acute myeloid leukaemia, Chronic myelogenous leukaemia, Hairy cell leukaemia, Hodgkin lymphoma, Monoclonal gammopathies, Myeloma, Myelodysplastic syndromes, Non-Hodgkin lymphoma			Solid tumours: Breast, Gastrointestinal, Genitourinary, Lung, Pheochromocytoma, Osteosarcoma, Ovarian, Thyroid		
Medications	Antibiotics: Azathioprine, Doxycycline, Clindamycin, Minocycline, Nitrofurantoin, Trimethoprim-Sulfamethoxazole	Antihypertensives: Hydralazine	Antineoplastics: Bortezomib, Ibrutinib, Imatinib, Lenalidomide	Nonsteroidal anti-inflammatory drugs	Diuretics: Frusemide	Antipsychotic: Lithium
Infections	Bacterial: Helicobacter pylori, Mycobacterium chelonae, Salmonella, Staphylococcus, Yersinia enterocolitica, Tuberculous mycobacteria		Fungal: Coccidiomycosis, Sporotrichosis	Viral: Cytomegalovirus, Hepatitis A, Hepatitis B, Human immunodeficiency virus (HIV), Parvovirus b19		
Systemic disorders	Behçet's disease		Inflammatory disease: Crohn’s disease, Ulcerative colitis	Rheumatoid arthritis	Sjögren's syndrome	Systemic lupus erythematosus
Miscellaneous	Erythema nodosum		Polycythaemia vera	Sarcoidosis		Thyroiditis

Treatment 

**Figure 2:**
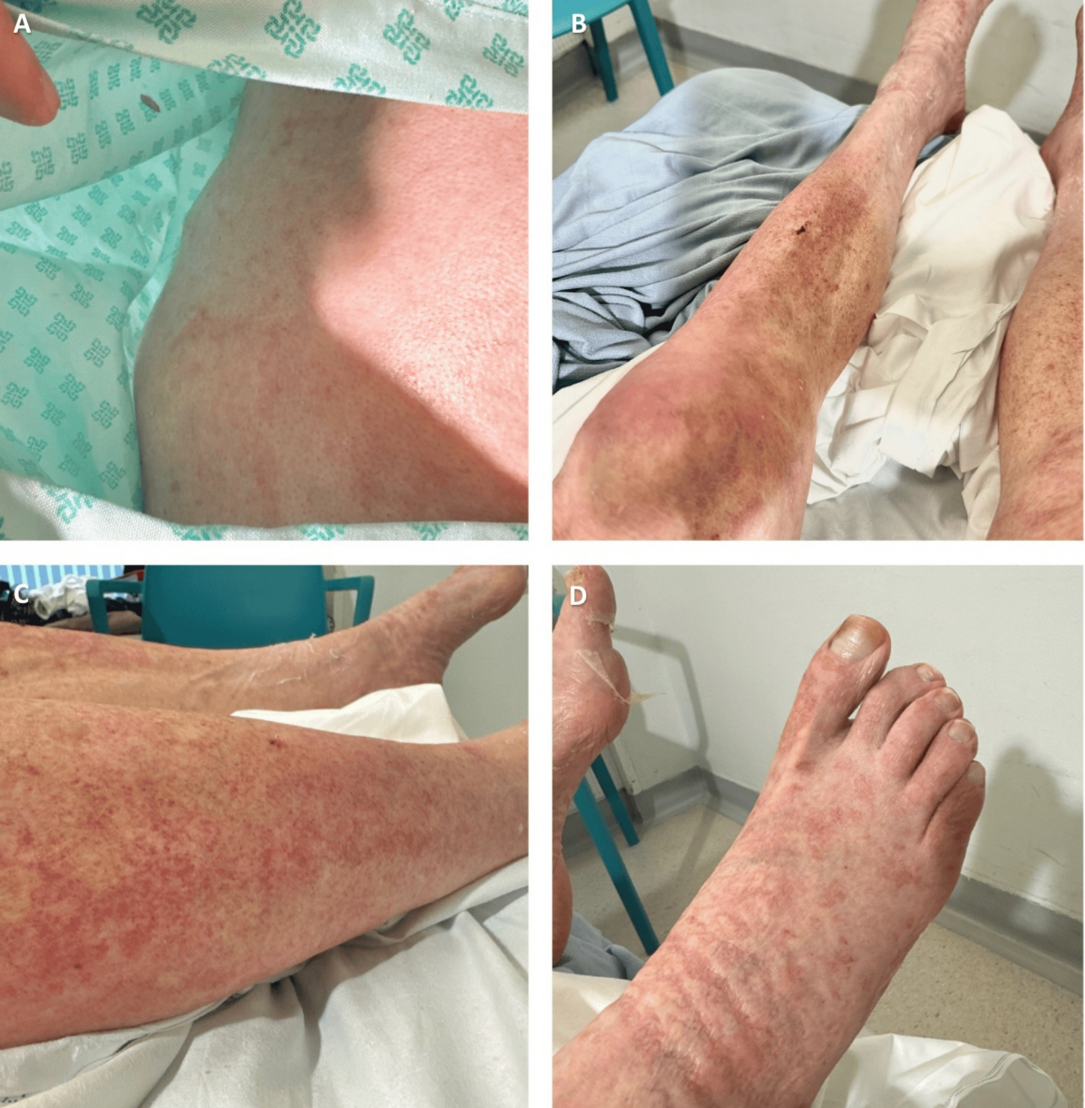
Fourteen days following the initiation of high-dose corticosteroids Significant improvement of the cutaneous manifestations, including flattening of nodules. (A) lower back and (B-D) lower limbs.

**Figure 3:**
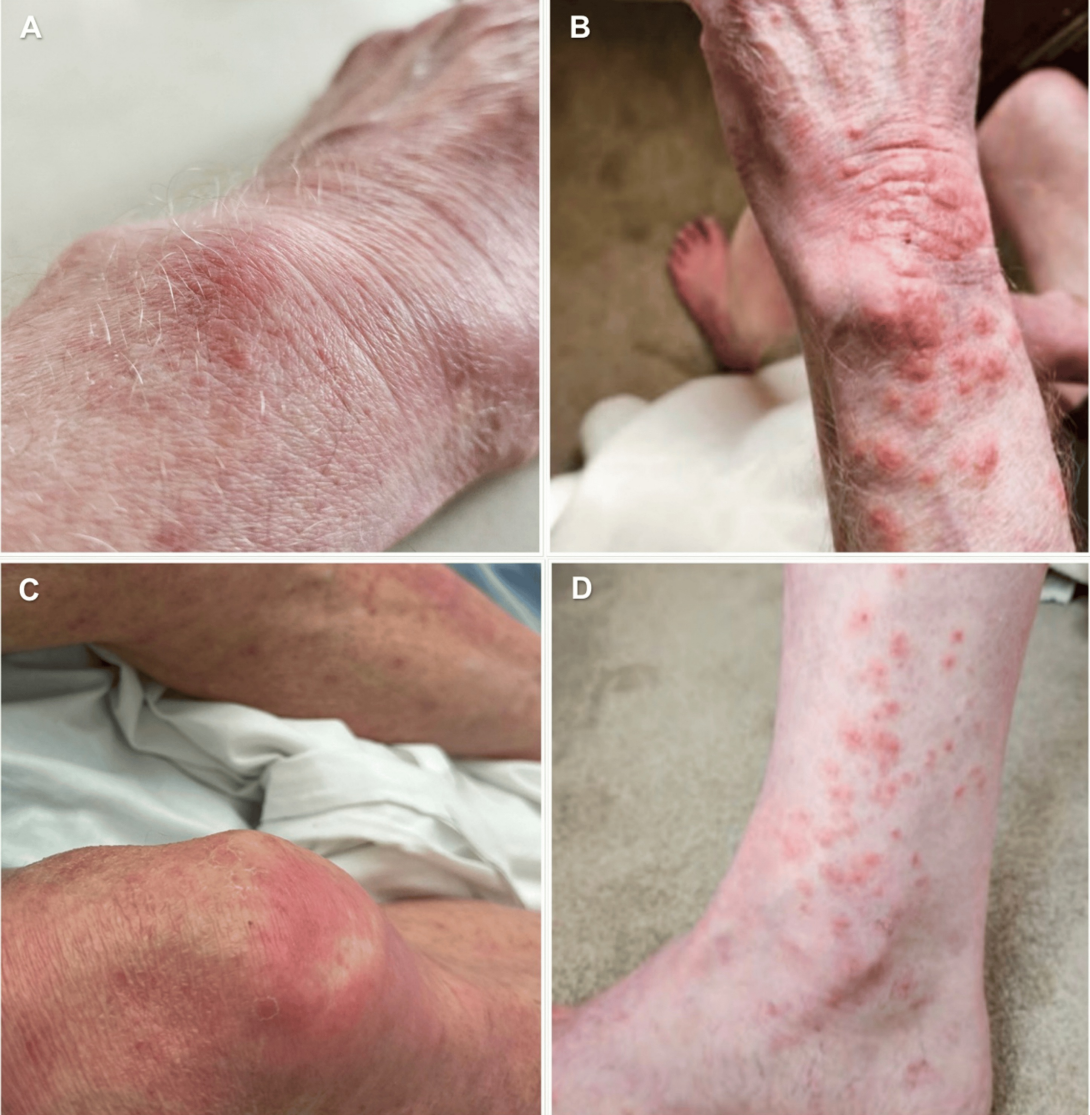
Twenty-five days after treatment with high-dose corticosteroids Flattening of nodules, residual desquamative changes. No active lesions. (A) dorsal of the left hand, (B) left forearm, and (C-D) lower limbs.

Following confirmation of SS, the prednisolone dose was increased to 40 mg daily. Within a few days, the CRP and neutrophil count improved to near-normal levels. This was coupled with a significant improvement in the disseminated body rash. After 14 days of high-dose steroid therapy, the patient improved back to his baseline with a marked improvement in his rash (Figure 2). By day 25, his skin rash had all but resolved with only residual desquamative changes and xerosis present (Figure 3). He was discharged for outpatient clinic review with a plan for gradual tapering of prednisolone over six weeks.

Outcome and follow-up

Ten days following discharge, the patient was reviewed in the clinic. He was well, and only residual desquamative skin lesions remained. Laboratory blood testing showed continued down-trending of inflammatory markers and acute phase reactants. The bone marrow biopsy revealed hypercellular changes with granulocytic hyperplasia and dyshaemopoiesis, consistent with a diagnosis of myelodysplastic syndrome (MDS). He is now receiving ongoing care from the haematology team as an outpatient.

## Discussion

SS was first described in 1964 by Dr. Robert Douglas Sweet and is also known as acute febrile neutrophilic dermatosis. It is an uncommon inflammatory skin condition that more commonly affects females than males and typically begins between the ages of 30 and 60 years. It is characterised by the rapid appearance of painful, well-defined plaques or nodules and constitutional symptoms including fever, myalgia, and fatigue. Extracutaneous involvements such as episcleritis, conjunctivitis, arthritis, and systemic dissemination leading to myocarditis or meningitis have all been reported [5].

In 1994, Von den Driesch [6] modified SS diagnostic criteria to include the presence of an abrupt disseminated tender or painful skin rash with histopathological evidence of neutrophilic dermal predominance as the two components of the major criteria. The minor criteria included the presence of fever >38°C, a preceding inflammatory disease, infection, haemoproliferative or solid organ malignancy, elevated inflammatory markers with neutrophilic predominance, and positive response to systemic corticosteroids [1]. Ten years later in 1996, Walker et al. [7] proposed new criteria for drug-induced SS that incorporated a temporal relationship between initiation and cessation of symptoms upon introducing and withdrawing the proposed offending agent.

First-line treatments for SS include local and systemic corticosteroids and, less commonly, potassium iodide or colchicine. Second-line agents include cyclosporine and dapsone. The effectiveness of medications with different mechanisms of action reflects the role of both the adaptive and innate cells in the pathogenesis of SS [5].

Drug-induced SS has been primarily linked to anti-cancer medications including azacytidine, decitabine, all-trans retinoic acid, and pomalidomide. These drugs are believed to cause an idiosyncratic halt in granulocyte maturation in the bone marrow, leading to a compensatory increase in granulocyte colony-stimulating factor levels [8]. In some cases, SS can resolved spontaneously. However, if drug-induced, improvement is facilitated by the withdrawal of the causative agent. Additionally, isolated case reports have observed improvement in both SS and associated haematologic disorders with malignancy-directed therapies, including the use of interferon-alpha [9].

Nearly 21% of SS cases are associated with underlying malignancies. Approximately 80% of these are haematological and include acute myeloid leukaemia (AML) and MDS, which can progress to AML. In cases of chronic lymphocytic leukaemia or multiple myeloma, SS can manifest as a paraneoplastic condition [10]. Importantly, within the context of an underlying haematological malignancy, SS skin manifestations can predate the diagnosis of malignancy and can signal the presence of cancer in previously undiagnosed individuals or indicate a recurrence in those with a prior history [11].

SS has fundamentally been regarded as a cutaneous marker of systemic disease, and it is not commonly associated with IE. Nayak et al. reported a case of Staphylococcal rheumatic mitral valve IE and SS, wherein there was no improvement in the rash with intravenous antibiotics [12]. The patient was subsequently begun on systemic corticosteroids with rapid improvement in manifestations of SS. Therefore, it would appear that the syndrome is not initiated by the IE but that it shares a concurrent pathogenic mechanism. Our patient presented in a somewhat similar fashion, albeit with predisposing risk factors for IE. These included the presence of a permanent pacemaker, recent valve replacement surgery, and generalized systemic symptoms. Despite this, a diagnosis of IE was rapidly and effectively excluded with the aid of negative blood cultures and cardiac imaging [2].

## Conclusions

SS should be considered when there is an abrupt onset of painful erythematous plaques or nodules and fever. In the presence of a new onset rash, there is a low threshold for excluding IE, particularly where a patient has active cardiac risk factors.

Additionally, SS should be considered a cutaneous marker of systemic disease, including infection and malignancy, and it can predate the diagnosis of malignancy. In terms of aetiology, SS can also be iatrogenic and pharmacotherapy related. An early skin biopsy is encouraged to expedite diagnosis and treatment. Corticosteroids should remain the cornerstone of treatment.
